# Risk factors for postoperative delirium in patients with Stanford type A aortic dissection: a systematic review and meta-analysis

**DOI:** 10.1186/s13019-024-02485-5

**Published:** 2024-01-22

**Authors:** Shan Lu, Yi Jiang, Fangfang Meng, Xiaoli Xie, Dongjin Wang, Yunyan Su

**Affiliations:** 1 Department of Thoracic and Cardiovascular Surgery, Nanjing Drum Tower Hospital, Affiliated Hospital of Medical School, Nanjing University, 321 Zhongshan Road, Nanjing, 210008 Jiangsu China; 2 Department of Cardiovascular Surgery, Nanjing Drum Tower Hospital, Chinese Academy of Medical Science & Peking Union Medical College, Nanjing, 210008 China; 3 Nanjing University, Nanjing, 210008 China

**Keywords:** Aortic dissection, Delirium, Risk factor, Meta-analysis

## Abstract

**Background:**

Delirium is a common postoperative complication among patients who undergo Stanford Type A aortic dissection (TAAD). It is associated with increased mortality, as well as other serious surgical outcomes. This study aimed to analyze the risk factors for delirium in TAAD patients.

**Methods:**

Pubmed, Web of science, Embase, the Cochrane Library and CINAHL were searched by computer to collect literatures on risk factors for postoperative delirium (POD) after TAAD. The retrieval period was from the establishment of the database to September 2022. After literature screening, two reviewers independently assessed the quality of the included studies using the Newcastle–Ottawa Scale (NOS). Data were extracted according to standard protocols, and then meta-analysis was performed using Revman 5.3 software.

**Results:**

A total of 9 articles, comprising 7 case–control studies and 2 cohort studies, were included in this analysis. The sample size consisted of 2035 patients. POD was associated with increased length of ICU stay (MD 3.24, 95% CI 0.18–6.31, *p* = 0.04) and length of hospital stay (MD 9.34, 95% CI 7.31–11.37, *p* < 0.0001) in TAAD patients. Various perioperative risk factors were identified, including age (MD 4.40, 95% CI 2.06–6.73, *p* = 0.0002), preoperative low hemoglobin levels (MD − 4.44, 95% CI − 7.67 to − 1.20, *p* = 0.007), body mass index (MD 0.92, 95% CI 0.22–1.63, *p* = 0.01), history of cardiac surgery (OR 3.06, 95% CI 1.20–7.83, *p* = 0.02), preoperative renal insufficiency (OR 2.50, 95% CI 1.04–6.04, *p* = 0.04), cardiopulmonary bypass (CPB) duration (MD 19.54, 95% CI 6.34–32.74, *p* = 0.004), surgery duration (MD 44.88, 95% CI 5.99–83.78, *p* = 0.02), mechanical ventilation time (SMD 1.14, 95% CI 0.34–1.94, *p* = 0.005), acute physiology and chronic health evaluation (APACHE II) score (MD 2.67, 95% CI 0.37–4.98, *p* = 0.02), postoperative renal insufficiency (OR 2.82, 95% CI 1.40–5.68, *p* = 0.004), electrolyte disturbance (OR 6.22, 95% CI 3.08–12.54, *p* < 0.0001) and hypoxemia (OR 3.56, 95% CI 1.70–7.44, *p* = 0.0007).

**Conclusions:**

POD can prolong ICU stay and hospital stay in TAAD patients. This study identified a number of risk factors for POD after TAAD, suggesting the possibility of early identification of high-risk patients using relevant data.

## Introduction

Aortic dissection (AD) refers to the pathological change where blood enters the media through the intimal tear and extends along the long axis of the aorta, peeling off the aortic wall and resulting in the separation of true and false lumens. The disease’s progress leads to the symptoms and signs of the corresponding organ blood supply disorder [1]. Stanford Type A aortic dissection (TAAD) has a high in-hospital mortality rate of 12.7–27.7% [2]. The mortality rate increases by 1–2% per hour in early untreated patients, with a mortality rate as high as 60–70% within one week [3]. Emergency surgery is currently the most effective treatment method, and the postoperative mortality rate has considerably decreased in recent years. However, patients are prone to neurological dysfunction after surgery because of the complexity of the operation, long operation time, and impact on brain tissue perfusion [4, 5].

Postoperative delirium (POD) is one of the common neurological complications in TAAD patients following surgery. It is an acute and reversible state of confusion characterized by fluctuating consciousness disorder, inattention and thinking disorder [6]. The incidence of POD in AD is approximately 32.5–52.0% [2]. Delirium leads to prolonged mechanical ventilation time and ICU stay, increased postoperative readmission possibility. It also increases the psychological pain of caregivers, economic burden, and the work pressure on medical teams [7, 8]. Furthermore, the occurrence of POD is closely related to increased postoperative mortality, higher medical expenses, long-term cognitive impairment, and decline in quality of life, leading to poor prognosis of patients [9, 10]. Early screening and management of delirium can reduce hospital mortality and the incidence of postoperative complications; it can also reduce the care burden of caregivers and the treatment and nursing pressure on medical and nursing teams [11–13]. As a result, it is vital to identify its risk factors, as many scholars have analyzed the risk factors of POD in TAAD patients. Common risk factors include cerebrovascular disease history, intubation time, surgery duration, renal insufficiency and hypoxia, etc.[14, 15]. However, the research results are controversial. For instance, Lv et al. [14] confirmed that age was a high-risk factor for POD, while Liu et al. [15] reported that age was not an independent risk factor for POD in TAAD patients. This controversial conclusion has brought some confusion in determining the high-risk factors for POD. Therefore, the purpose of this study is to identify risk factors by integrating and analyzing the existing literature on POD in TAAD patients This will help accurately identify high-risk groups and provide an evidence-based foundation for clinical medical staff to develop targeted perioperative treatment and management measures.

## Methods

### Search strategy

This study was reported by referring to the statement of the preferred reporting items for systematic reviews and meta-analyses (PRISMA) [16]. Before the initial search, it was registered with the international prospective register of systematic reviews (PROSPERO) database (Registration Number: CRD42021232401). We searched the data sources, namely, PubMed, Web of Science, Embase, the Cochrane Library, and Cumulative Index to Nursing and Allied Health Literature (CINAHL), from the establishment of each database to September 2022. In addition, the grey literature information sources, including ProQuest Medical Library, PROSPERO database, U.S. National Library of Medicine, and Chinese Clinical Trial Registry, were searched up to August 2022. The language was limited to Chinese and English. The following combination of medical subject headings and keywords were used for searching: “aortic dissection” or “Stanford type A aortic dissection” or “TAAD” or “aortic aneurysm” or “dissecting aneurysm”, “delirium” or “delirium syndrome” or “phrenitis” or “deliration” or “ideosynchysia” and “risk factor*” or “influence factor*” or “related factor*” or “associate factor*” or “predictor*” or “factor*”. The retrieval strategy was jointly formulated by medical librarians. The references of the included literature were searched manually to supplement the acquisition of relevant literature.

### Selection criteria

EndNote X9 software was used for literature management. The relevant data were extracted and recorded in the form of a database. For the literature with incomplete data, we contacted the original authors for the supplement. The articles included in the study must meet the following inclusion criteria: (1) The case–control study or cohort study analyzing the risk factors of POD in patients with TAAD has been published. (2) The subjects were TAAD patients who received surgical treatment and were more than 18 years old. (3) The outcome indicators included the risk factors of delirium, the control group of patients without delirium, and the provided data on the incidence of delirium. Articles with unavailable full text for abstract publication, those with data in the original study that could not be extracted or transformed, and repeated publications were excluded. Two researchers (LS and MFF) screened the literature according to the inclusion and exclusion criteria. They also extracted the data and cross-checked them. The following variables were extracted from the included studies: author, publication year, study design, sample size, inclusion and exclusion criteria of population, delirium criteria, assessment instrument, screening frequency, and study quality score. In case of disagreement, the third researcher (JY) was asked to assist in judgment.

### Study quality assessments

Two researchers (LS and MFF) evaluated the quality of the included studies independently by using the Newcastle–Ottawa Scale (NOS) [17]. NOS consists of two subscales, the quality evaluation form of a case–control study and a cohort study, which has three categories and eight items for studying subject selection, intergroup comparability, and outcome or exposure factor measurement. The total score of NOS is 9 points, with ≤ 4 points as low-quality research, 5 – 6 points as medium-quality research, and ≥ 7 points as high-quality research. After the evaluation, the two researchers cross-checked the evaluation results. If the scoring results were inconsistent, the third researcher (JY) was consulted or discussed for judgment.

### Data analysis and synthesis

Revman 5.3 software (The Cochrane Collaboration, Copenhagen, Denmark) was used to perform a meta-analysis on the extracted data. Q test was used for studying heterogeneity assessment. If I^2^ ≤ 50%, homogeneity existed between the studies, and the fixed effect model was used for meta-analysis. If I^2^ > 50%, heterogeneity among studies was indicated. Sensitivity analysis was performed to ensure the reliability and stability of the studies, and the random effects model was used for meta-analysis. *P* ≤ 0.05 was considered to be statistically significant. The cumulative effect amount for dichotomous variables was presented as odds ratio (OR). Mean difference (MD) was used for continuous variables where outcome indicators used the same measurement methods and units, whereas standard mean difference (SMD) was used for continuous result indicators with different measurement methods or units. All outcome indicators were given 95% confidence interval (CI). When the number of articles included in a single risk factor analysis was ≥ 7, a funnel plot was used to analyze publication bias.

## Results

### Study selection

**Fig. 1:**
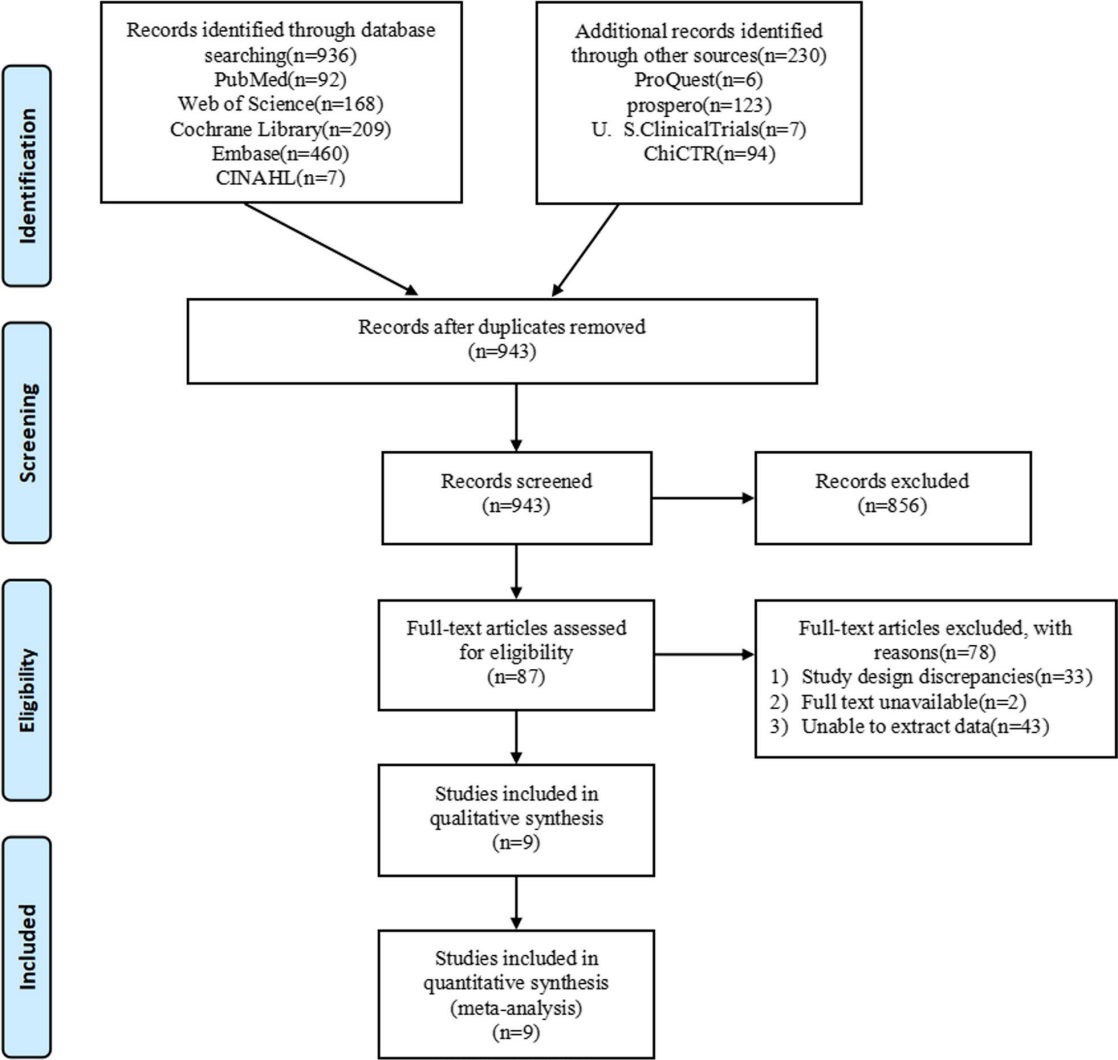
Preferred reporting items for systematic reviews and meta-analyses (PRISMA) flow diagram

Initially, 1166 articles were obtained after retrieval, and 943 articles remained after EndNote X9 was introduced to remove duplicates. 87 articles were read after reading the titles and abstracts and removing the irrelevant ones. According to the inclusion and exclusion criteria, the study design of 33 articles was inconsistent, while the full text of 2 articles could not be obtained, and the data of 43 articles could not be extracted. Finally, nine articles [14, 15, 18–24] were included in the study. The literature screening flowchart is shown in Fig. 1.

### Description of included studies and quality appraisal

Nine studies met the criteria of this study. The influencing factors of POD in patients undergoing TAAD were investigated. The literature included seven case control studies and two cohort studies, with a total of 2035 patients. Each study described the assessment tools and diagnostic criteria for delirium, including the Confusion Assessment Method for the ICU (CAM-ICU), the Diagnostic and Statistical Manual of Mental Disorders, and Fifth Edition (DSM-5). The NOS evaluation scores of all the works were above 7, indicating high quality. The general information and the article quality evaluation results of the included articles are shown in Table 1.

**Table 1:** General characteristics of the included studies

Study	Design	Population	Sample size	POD assessment	Diagnosis frequency and duration	POD incidence (%)	Study quality score
Fang 2016 [18]	Case–control study	Inclusion criteria: underwent TAAD surgery Exclusion criteria: (1) age < 18 or > 65; (2) delirium, psychosis, mental retardation, and low cognitive level before surgery; (3) stroke before surgery; (4) death during surgery; (5) lack of consciousness after surgery; (6) ICU stay < 24 h; (7) incomplete data collection	335	CAM-ICU	Start 24 h after surgery, every 8 h until the 10th postoperative day or leaving the ICU	50.4	8
Song 2016 [19]	Case–control study	Inclusion criteria: underwent TAAD surgery and were followed in the cardiac care unit Exclusion criteria: (1) delirium or mental illness diagnosis before surgery; (2) failure to recover from postoperative coma; (3) age < 18; (4) perioperative death; (5) ICU stay < 24 h; (6) incomplete data collection	148	CAM-ICU	3 times a day (7:00, 15:00, 23:00)	31.1	8
Liu 2017 [15]	Case–control study	Inclusion criteria: TAAD patients after Sun’s procedure in the cardiac surgical intensive care unit, with or without aortic valve replacement Exclusion criteria: (1) preoperative mental disease diagnosis; (2) inability to wake from surgery; (3) death during or within 24 h of surgery; (4) incomplete data collection	100	CAM-ICU	Every 4 h postoperatively until patients left the ICU	34	8
Shi 2019 [20]	Case–control study	Inclusion criteria: underwent Sun operation in the cardiac surgery ICU Exclusion criteria: death or discharge from hospital	148	CAM-ICU	NS	45.95	8
Lin 2020 [21]	Cohort study	Inclusion criteria: (1) age 18 – 75 years; (2) consent and volunteering to participate in this study Exclusion criteria: (1) brain injury history; (2) congenital deaf-mute; (3) postoperative ICU stay < 24 h; (4) continuous deep sedation with propofol and remifentanil after surgery	280	CAM-ICU	From the 1st day after surgery lasting until the 5th day after surgery	37.86	8
Cai 2020 [22]	Case–control study	Inclusion criteria: the diagnosis included in TAAD was CT angiography Exclusion criteria: mental disorders	301	CAM-ICU	NS	24.25	9
Lin 2021 [23]	Cohort study	Inclusion criteria: underwent TAAD surgery Exclusion criteria: less than 48 h stay in ICU after the operation, transcranial trauma history, congenital deafness or schizophrenia, epilepsy before surgery, using glucocorticoids for a long time, remaining in a coma	257	CAM-ICU	From 8:00 to 11:00, 15:00 to 17:00, and 20:00 to 23:00 on the first day after surgery, until delirium occurred or the patient was transferred out of ICU	40.08	9
Lv 2021 [14]	Case–control study	Inclusion criteria: (1) age > 18 years; (2) surgical repair combined with open triple-branched stent graft placement; (3) signed informed consent Exclusion criteria: (1) prior neurological or psychiatric diseases; (2) liver cirrhosis and uremia; (3) preoperative stroke or brain malperfusion; (4) preoperative shock or hemodynamic instability due to cardiac tamponade; (5) liver enzymes greater than four times the baseline; (6) hearing or visual impairment; (7) comatose after surgery or died within 24 h after the surgery; (8) extracorporeal membrane oxygenation therapy	221	CAM-ICU	After the first 3 postoperative days, delirium was evaluated twice daily in ICU or the general ward	14.03	8
Li2022 [24]	Case–control study	Inclusion criteria: (1) TAAD; (2) no history of delirium; (3) underwent surgical treatment Exclusion criteria: (1) death during hospitalization; (2) withdrawal of treatment due to deterioration of primary disease; (3) cerebrovascular sequelae; (4) history of mental illness, alcohol, or drug abuse	245	DSM-5	NS	20.82	8

*POD* postoperative delirium, *CAM-ICU* Confusion Assessment Method for the Intensive Care Unit; *DSM-5* diagnostic, statistical manual of mental disorders, and fifth edition

### Meta-analysis for outcome measures

**Fig. 2:**
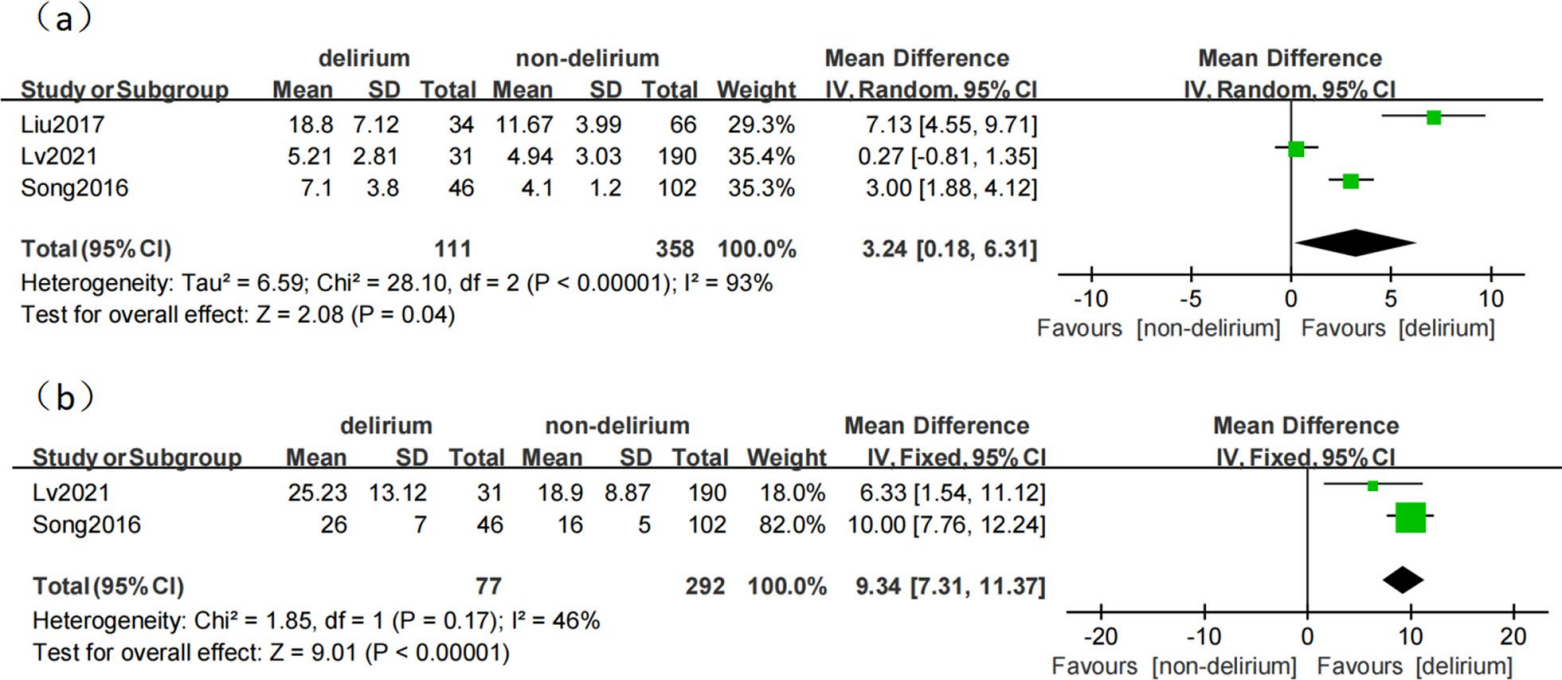
**a** The association between duration of ICU stay and delirium. **b** The association between hospital stay and delirium

We found that the incidence of POD after TAAD was between 14.03 and 50.4% (Table 1). In most studies, the patients were evaluated for delirium from the day after the surgery until they were transferred out of the ICU or the general ward. According to the data analysis, a statistically significant association of POD existed between the increased duration of ICU stay (MD 3.24, 95% CI 0.18–6.31, *p* = 0.04; Fig. 2) and the prolonged hospital stay (MD 9.34, 95% CI 7.31–11.37, *p* < 0.0001, Fig. 2). We also determined 12 significant influencing factors (*p* < 0.05) divided into preoperative, intraoperative, and postoperative factors. However, due to differences in sample size, individual differences in the study population, and variations in delirium assessment and diagnostic methods among the different studies, heterogeneity exists among some studies.

### Preoperative data

#### Age

**Fig. 3:**
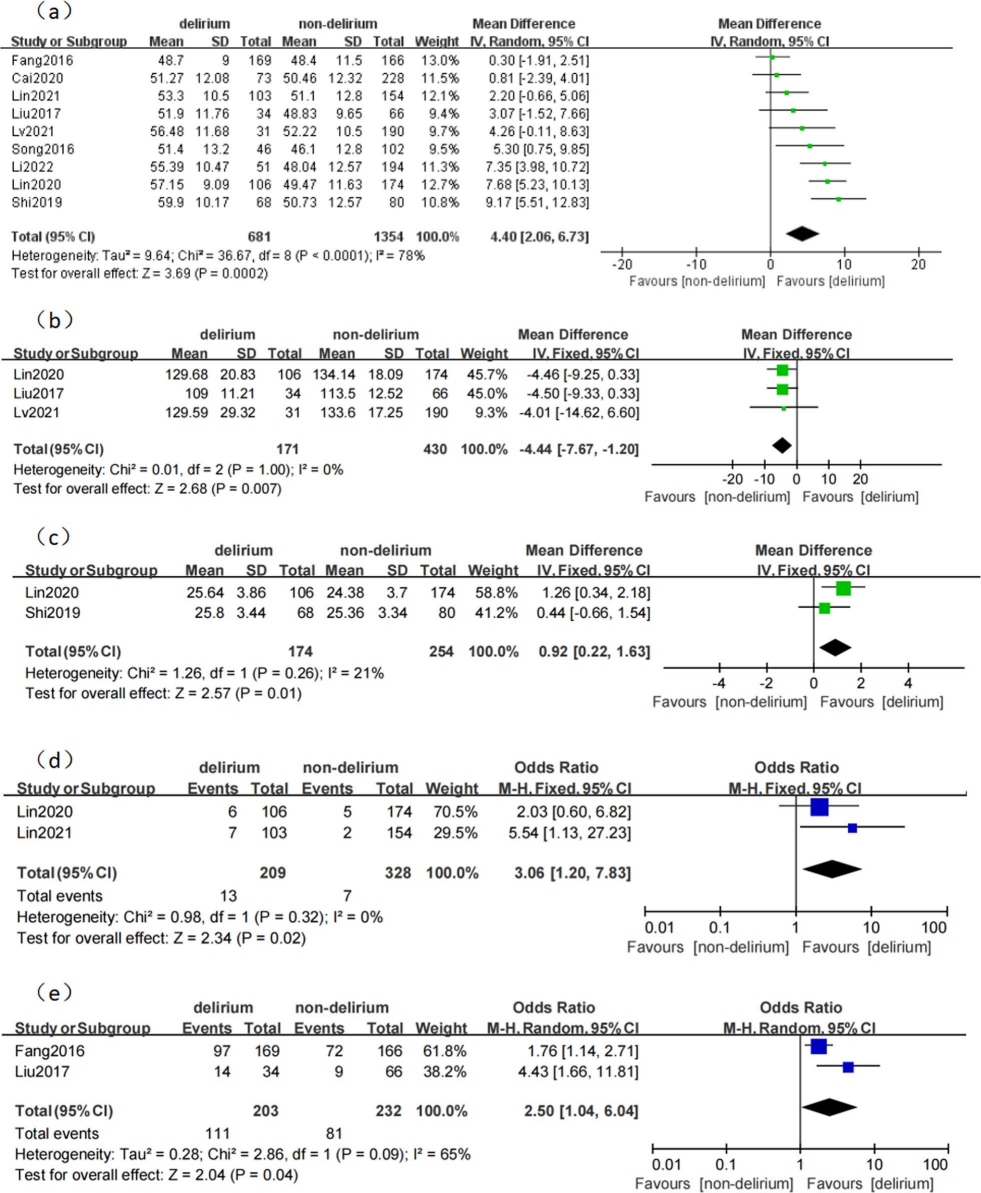
**a** The association between age and delirium. **b** The association between preoperative low hemoglobin levels and delirium. **c** The association between body mass index (BMI) and delirum. **d** The association between history of cardiac surgery and delirium. **e** The association between preoperative renal insufficiency and delirium

Nine studies [14, 15, 18–24] reported the influence of age on POD after TAAD. The studies showed heterogeneity (*p* < 0.0001; I^2^ = 78%). The results of the random effect model showed that age was the risk factor for delirium after TAAD (MD 4.40, 95% CI 2.06–6.73, *p* = 0.0002; Fig. 3).

#### Preoperative low hemoglobin levels

Three studies [14, 15, 21] reported the effect of preoperative low hemoglobin levels on POD after TAAD. The studies had no heterogeneity (*p* = 1.00; I^2^ = 0%). The results of the fixed effect model showed that preoperative low hemoglobin levels were the risk factor for delirium after TAAD (MD − 4.44, 95% CI − 7.67 to − 1.20, *p* = 0.007; Fig. 3).

#### Body mass index

Two studies [20, 21] reported the effect of body mass index (BMI) on POD after TAAD. The studies had no heterogeneity (*p* = 0.26; I^2^ = 21%). The results of the fixed effect model showed that BMI was the risk factor for delirium after TAAD (MD 0.92, 95% CI 0.22–1.63, *p* = 0.01; Fig. 3).

#### History of cardiac surgery

Two studies [21, 23] reported the effect of cardiac surgery history on POD after TAAD. The studies had no heterogeneity (*p* = 0.32; I^2^ = 0%). The results of the fixed effect model showed that cardiac surgery history was the risk factor for delirium after TAAD (OR 3.06, 95% CI 1.20–7.83, *p* = 0.02; Fig. 3).

#### Preoperative renal insufficiency

Two studies [15, 18] reported the effect of preoperative renal insufficiency on POD after TAAD. The studies showed heterogeneity (*p* = 0.09; I^2^ = 65%). The results of the random effect model showed that a high creatinine level was the risk factor for delirium after TAAD (OR 2.50, 95% CI 1.04–6.04, *p* = 0.04; Fig. 3).

### Intraoperative data

#### Cardiopulmonary bypass duration

**Fig. 4:**
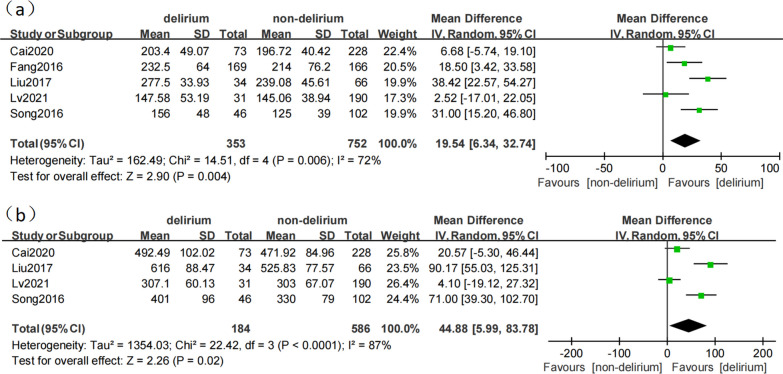
**a** The association between cardiopulmonary bypass (CPB) duration and delirium. **b** The association between surgery duration and delirium

Five studies [14, 15, 18, 19, 22] reported the effect of Cardiopulmonary bypass (CPB) duration on POD after TAAD. The studies showed heterogeneity (*p* = 0.006; I^2^ = 72%). The results of the random effect model showed that CPB duration was the risk factor for delirium after TAAD (MD 19.54, 95% CI 6.34–32.74, *p* = 0.004; Fig. 4).

#### Surgery duration

Four studies [14, 15, 19, 22] reported the effect of surgery duration on POD after TAAD. The studies showed heterogeneity (*p* < 0.0001; I^2^ = 87%). The results of the random effect model showed that surgery duration was the risk factor for delirium after TAAD (MD 44.88, 95% CI 5.99–83.78, *p* = 0.02; Fig. 4).

### Postoperative data

#### Mechanical ventilation time

**Fig. 5:**
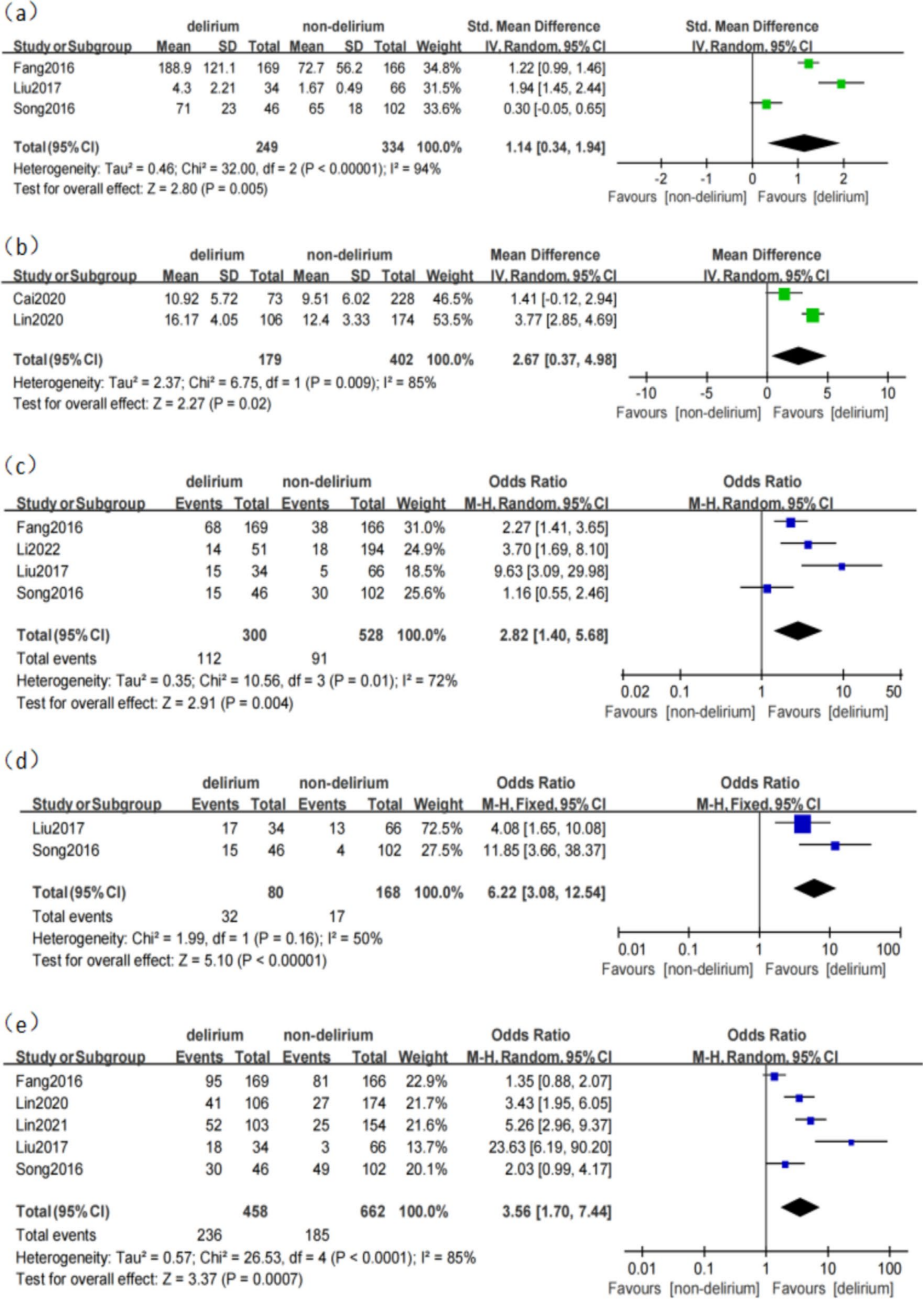
**a** The association between mechanical ventilation time and delirium. **b** The association between Acute physiologyand chronic healthevaluation (APACHE II) score and delirium. **c** The association between postoperative renal insufficiency and delirium. **d** The association between electrolyte disturbance and delirium. **e** The association between hypoxemia and delirium

Three studies [15, 18, 19] reported the effect of mechanical ventilation time on POD after TAAD. The studies showed heterogeneity (*p* < 0.0001; I^2^ = 94%). The results of the random effect model showed that mechanical ventilation time was the risk factor of delirium after TAAD (SMD 1.14, 95% CI 0.34–1.94, *p* = 0.005; Fig. 5).

#### Acute physiology and chronic health evaluation score

Two studies [21, 22] reported the effect of the Acute physiology and chronic health evaluation (APACHE II) score on POD after TAAD. The studies showed heterogeneity (*p* = 0.009; I^2^ = 85%). The results of the random effect model showed that the APACHE II score was the risk factor for delirium after TAAD (MD 2.67, 95% CI 0.37–4.98, *p* = 0.02; Fig. 5).

#### Postoperative renal insufficiency

Four studies [15, 18, 19, 24] reported the effect of postoperative renal insufficiency on POD after TAAD. The studies showed heterogeneity (*p* = 0.01; I^2^ = 72%). The results of the random effect model showed that renal insufficiency was the risk factor of delirium after TAAD (OR 2.82, 95% CI 1.40–5.68, *p* = 0.004; Fig. 5).

#### Electrolyte disturbance

Two studies [15, 19] reported the effect of electrolyte disturbance on POD after TAAD. Electrolyte disorder refers to metabolic disorder of serum sodium, potassium and magnesium. The studies showed there was no heterogeneity (*p* = 0.16; I^2^ = 50%). The results of the fixed effect model showed that electrolyte disturbance was the risk factor of delirium after TAAD (OR 6.22, 95% CI 3.08–12.54, *p* < 0.0001; Fig. 5).

#### Hypoxemia

Five studies [15, 18, 19, 21, 23] reported the effect of hypoxemia on POD after TAAD. The studies showed heterogeneity (*p* < 0.0001; I^2^ = 85%). The random effect model was used to combine. The results of the random effect model showed that hypoxemia was the risk factor for delirium after TAAD (OR 3.56, 95% CI 1.70–7.44, *p* = 0.0007; Fig. 5).

### Sensitivity analysis

Sensitivity analysis was carried out by means of conversion effect model, and the results showed no significant changes, as shown in Table 2, which indicating that the combined results of meta-analysis had a certain degree of stability and reliability.

**Table 2:** Comparison of results between random effect model and fixed effect model

Risk factor	Random effect model	Fixed effect model
	OR/MD (95%CI)	OR/MD (95%CI)
ICU stay	3.24 (0.18, 6.31)	2.04 (1.30, 2.79)
Age	4.40 (2.06, 6.73)	4.05 (2.99, 5.10)
Preoperative renal insufficiency	2.50 (1.04, 6.04)	2.04 (1.37, 3.02)
Cardiopulmonary bypass duration	19.54 (6.34, 32.74)	19.00 (12.18, 25.83)
Surgery duration	44.88 (5.99, 83.78)	35.32 (21.39, 49.24)
Mechanical ventilation time	1.14 (0.34, 1.94)	1.07 (0.89, 1.25)
Acute physiology and chronic health evaluation score	2.67 (0.37, 4.98)	3.15 (2.36, 3.93)
Postoperative renal insufficiency	2.82 (1.40, 5.68)	2.45 (1.75, 3.43)
Hypoxemia	3.56 (1.70, 7.44)	2.69 (2.07, 3.48)

### Publication bias

**Fig. 6:**
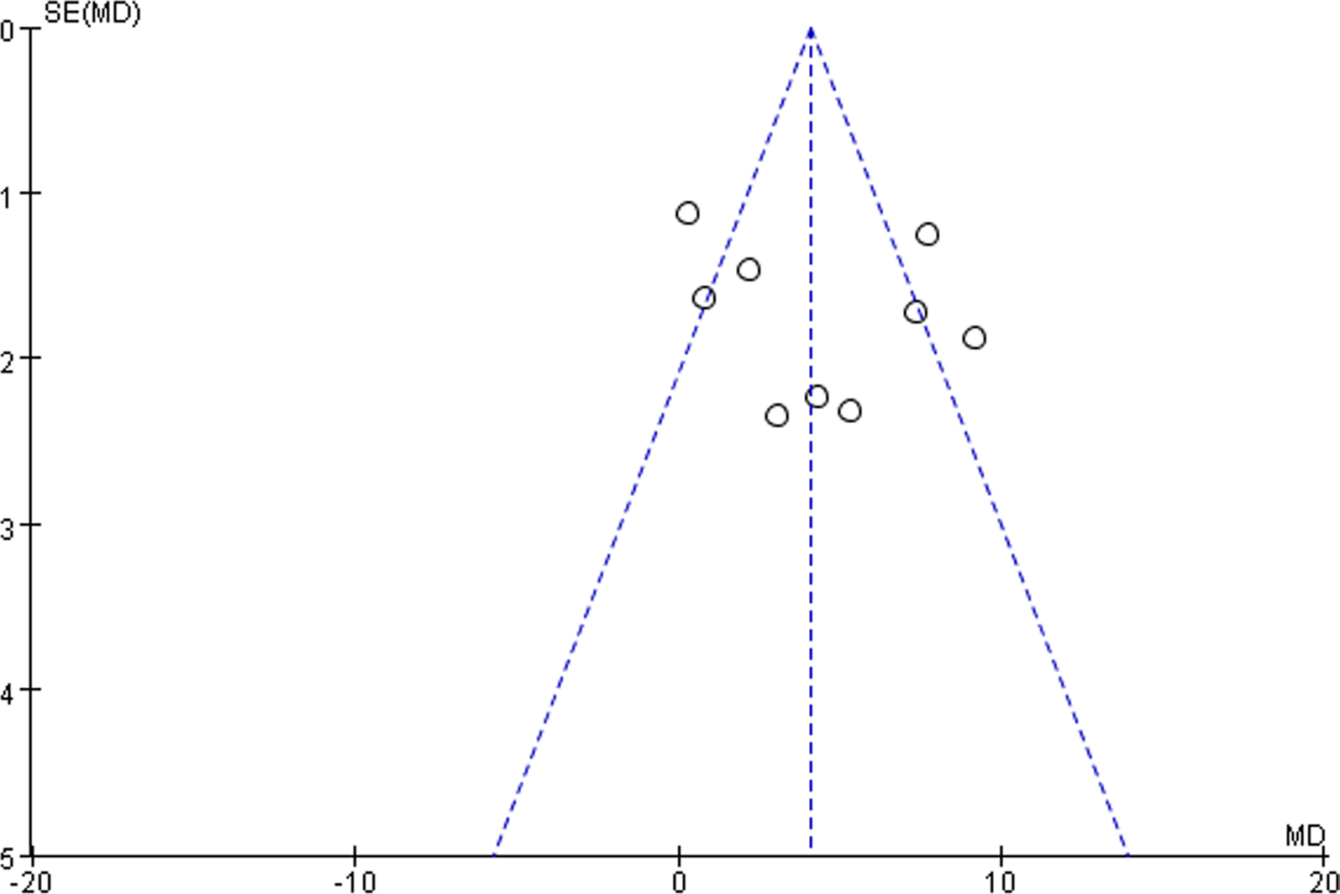
Funnel plot results of risk factors for age

The funnel plot analysis of literature on the risk factors for age showed that the distribution of each literature was basically symmetrical, indicating a low publication bias (Fig. 6).

## Discussion

Delirium is a serious and complicated neurological complication in patients with TAAD. Cai et al. [22] found that patients with POD had longer ICU stays by 4.3 days and hospital stays by 5.08 days, respectively, when compared to those without delirium. Therefore, it is of great significance to identify and intervene in the risk factors of POD in patients with TAAD. This study systematically evaluated the risk factors for delirium in TAAD patients through meta-analysis, and ultimately included 9 studies. The literature quality was high, and the meta-analysis results were relatively reliable. The present study found that the incidence of POD fluctuated from 14.03 to 50.4%, and POD was associated with the patient’s ICU stay and hospitalization time. In this meta-analysis, we found 12 influencing factors.

The results of this study showed that age was one of the risk factors for POD in TAAD patients. Katarzyna et al. [25] reported that the incidence of delirium after cardiac surgery was 21.4% in patients aged over 65 years old, while 33.5% in patients over 80 years old. Mu et al. [26] found that the incidence of delirium increased by 8% with each year of age. The possible reasons are the continuous deterioration of brain metabolism and central nervous function with the increase in age, the decline of the self-regulation function of cerebral blood flow, and the easy occurrence of insufficient cerebral perfusion during surgery, resulting in damage to the neurotransmitter function [27]. In addition, elderly patients are mostly accompanied by chronic diseases. Their basic cognitive level is also affected to varying degrees, thereby increasing the risk of POD in the elderly.

Preoperative low hemoglobin levels were regarded as a risk factor for POD in TAAD patients. Preoperative low hemoglobin levels may lead to neurotransmitter imbalances, disruption of the brain barrier, and neuroinflammation due to decreased oxygen carrying capacity of the blood and insufficient oxidative metabolism of the brain. This can result in various levels of mental symptoms and even delirium [28]. Visser et al. suggested that preoperative low hemoglobin levels may be associated with an increased risk of delirium [29]. Therefore, the anemia status of TAAD patients with preoperative low hemoglobin levels should be corrected in time. Monitoring and prevention of POD should also be carried out.

BMI, an important index reflecting human nutrition and health status, is considered one of the risk factors for POD in TAAD patients. An abnormal BMI (overweight or obesity) may be linked to hyperlipidemia, which can cause problems with brain microcirculation, as well as ischemia and hypoxia in the brain tissue, which can result in cognitive dysfunction [30]. In addition, obstructive sleep apnea syndrome is also more severe in obese people, which causes transient hypoxia, inflammation, and oxidative stress in the body. This worsens the condition of nervous system malfunction [31].

The findings of the present study indicated that history of cardiac surgery is also a risk factor for POD in TAAD patients. Surgery is a serious trauma to the body, and repeated surgeries causes could induce a state of stress. This condition can lead to increased levels of adrenaline and norepinephrine, accelerated cerebral blood flow, increased oxygen consumption, norepinephrine–acetylcholine imbalance, and a series of neurological complications. Furthermore, stress can increase the activity of cholinesterase in the brain, enhance the metabolism of acetylcholine, and reduce the acetylcholine content, thereby inducing delirium [32].

This study found that renal insufficiency before and after surgery was a risk factor for POD in TAAD patients. The patient’s preoperative renal insufficiency may be due to the influence of dissection on kidney perfusion or the patient’s preoperative renal function. Acute renal failure is one of the common complications after TAAD operation, with an incidence rate ranging from 14 to 44% [33, 34]. Renal insufficiency can cause internal environment disorder, accumulation of toxic substances or inflammatory media, and aggravate tissue edema and brain edema. In addition, the disturbance of electrolyte and acid–base balance can directly affect the recovery of brain metabolism, further increasing the possibility of brain injury [35]. Therefore, renal protection in patients with TAAD is very important. Special attention should be paid to patients with renal insufficiency before surgery. Moreover, renal function monitoring should be strengthened after surgery. Once renal function deteriorates, hemofiltration therapy should be performed immediately to prevent irreversible damage to the whole body’s organs caused by metabolites and toxins.

CPB duration was associated with a high risk of POD in patients with TAAD. Liu et al. [15] pointed out that longer CPB durations lead to the release of more inflammatory mediators; this phenomenon can make the cerebral blood vessels contract, eventually inhibit cerebral blood flow, and impair brain function. Prolonged CPB may cause severe pulmonary complications, such as atelectasis, respiratory distress, and hypoxemia; it may also affect the blood supply to brain tissue and induce delirium [36]. On the one hand, the operation technique should be improved, CPB duration should be shortened, and the blood oxygen supply to the brain should be sufficient. On the other hand, postoperative monitoring should evaluate lung ventilation tolerance timely. Moreover, mechanical-assisted ventilation should be given when necessary to maintain the stability of ventilator parameters and hemodynamics of patients and avoid delirium induced by body ischemia and hypoxia.

This study found that longer operation time was one of the risk factors for POD in TAAD patients. Mu et al. [26] showed that the risk of delirium increased by 36% every hour of prolonged operation. Prolongation of operation time can extend anesthesia time correspondingly. Fentanyl, propofol and other anesthetic drugs act on cholinergic neuronal receptors for a long time, forming inhibition of the cholinergic nerve effect and easily causing delirium [37].

Mechanical ventilation was a risk factor for POD in patients with TAAD. Ely et al. [37] showed that delirium may occur in patients receiving mechanical ventilation; this finding is consistent with the present study. According to earlier research [38, 39], delirium occurred 20%–50% of the time in patients who did not receive mechanical ventilation and 60%–80% of the time in those who did. Delirium is an independent risk factor for offline difficulties and leads to prolonged mechanical ventilation [40]. Therefore, a cluster care plan, including daily wake-up and early functional exercise, can be developed to shorten the time of mechanical ventilation as much as possible to reduce the delirium occurrence further.

The results of this study showed that the APACHE II score was closely related to the POD occurrence in TAAD patients. This finding is consistent with the research conducted by Pei et al. [41]. The APACHE II score is a widely used and authoritative scoring system to evaluate disease severity. The severity of the patient's condition is inversely correlated with their APACHE II score. As the severity of a patient's condition increases, the length of their stay in the ICU after surgery also increases. During the postoperative stay in ICU, patients not only suffered from physical discomfort but also lacked the companionship of their families. Their anxiety and panic increased, leading to delirium.

Electrolyte disturbance was identified as a risk factor for POD in patients with TAAD. Xing et al. [42] pointed out that metabolic alkalosis affects the central nervous system, causing agitation, insanity and other manifestations. Patients with metabolic acidosis experience not only the effects on the central nervous system but also ion imbalance, thereby weakening heart contraction, decreasing the effective blood volume, and worsening brain dysfunction. Therefore, medical staff should actively correct patients’ acid–base imbalance and electrolyte disturbance to maintain the homeostasis of patients’ internal environment.

Hypoxemia was closely related to POD in TAAD patients. In patients with hypoxemia, the body’s oxygen consumption and supply are unbalanced. Moreover, the metabolic mode of brain cells changes easily under the hypoxia state, leading to nerve cell damage and brain edema. Chronic severe cerebral hypoxia can also affect acetylcholine levels, the electrical activity of brain tissue, sleep patterns and memory; it can also quickly result in delirium [43]. Therefore, medical staff should pay attention to the oxygen supply of TAAD patients after the operation and provide oxygen therapy to patients with hypoxemia after the operation to improve the brain oxygen supply and reduce the incidence of POD.

### Limitation

This study encountered some limitations. At first, the studies included had lower quality than randomized controlled trials. The majority of them were retrospective studies, which could have selection bias. Secondly, a comprehensive study of the elements that the risk of POD in TAAD patients was hampered by the minimal number of studies that could be included in the meta-analysis. Thirdly, the included studies were Chinese and English literature. The works in the literature meeting the inclusion criteria were all from Chinese regional studies, indicating some publication bias. At present, research- and evidence-based medicine must fully understand the risk factors for delirium following TAAD, because the evidence in this area is currently insufficient.

## Conclusion

POD can prolong the postoperative ICU stay and hospital stay in TAAD patients. The results of the present study show that age, preoperative hemoglobin levels, BMI, cardiac surgery history, preoperative renal insufficiency, CPB duration, surgery duration, mechanical ventilation time, APACHE II score, postoperative renal insufficiency, electrolyte disturbance and hypoxemia are the risk factors for POD in patients with TAAD. In clinical practice, identifying the risk factors of delirium in TAAD patients early is conducive to the early identification and prevention of delirium. According to the correctable factors in delirium risk factors, this study is expected to provide a reference for the formulation of effective intervention programs to reduce the incidence of delirium in TAAD patients further.

## Data Availability

All data used in this study were retrieved from public literature.
